# Aberrant Fear: Biological Underpinnings Relevant to Psychosis, Antipsychotic Drugs, and Psychotherapeutic Treatments, a Translational Approach

**DOI:** 10.3390/ijms27135681

**Published:** 2026-06-24

**Authors:** Benedetta Mazza, Licia Vellucci, Mariateresa Ciccarelli, Felice Iasevoli, Roberto Vitelli, Giuseppe De Simone, Carmine Tomasetti, Manami Fukutomi, Annarita Barone, Andrea de Bartolomeis

**Affiliations:** 1 Section of Psychiatry, Laboratory of Molecular and Translational Psychiatry, Unit of Treatment-Resistant Psychiatric Disorders, Department of Neuroscience, Reproductive Sciences and Dentistry, University School of Medicine of Naples Federico II, 80131 Naples, Italy; benedettamazza1996@gmail.com (B.M.);; 2 Section of Psychology, Department of Neuroscience, Reproductive Science and Dentistry, University Medical School of Naples Federico II, 80131 Naples, Italy; 3 National Health Service, Department of Mental Health, Alzheimer Center of Giulianova, ASL 4, 64021 Teramo, Italy; 4 UNESCO Chair on Health Education and Sustainable Development, University of Naples Federico II, 80131 Naples, Italy

**Keywords:** fear, schizophrenia, synapse, antipsychotics, psychotherapy, treatment-resistant psychosis

## Abstract

Fear is a transdiagnostic construct implicated in multiple psychiatric disorders, reflecting a partial dissociation between clinical phenotypes and underlying neurobiological mechanisms. Converging evidence suggests that aberrant fear processing plays a central role in cognitive and psychopathological models of psychosis. In this narrative review, we synthesize evidence on the neurobiological mechanisms of aberrant fear modulation in schizophrenia from a translational perspective, integrating findings from neuroimaging, preclinical models, pharmacological interventions, and psychotherapy. Schizophrenia is characterized by aberrant emotional processing and inappropriate neural responses to stimuli with reduced or absent objective salience, reflecting impaired discrimination of relevant environmental information. At the system level, evidence implicates dysregulation of cortico-limbic and salience-processing networks in altered fear learning, threat appraisal, and emotional prediction. Neurochemical findings indicate that dopamine–glutamate dysregulation and associated intracellular signaling pathways act as upstream modulatory mechanisms contributing to these network-level abnormalities. Therapeutic interventions, including antipsychotic drugs and psychotherapeutic approaches, partially modulate these systems, although effects remain heterogeneous. Overall, the evidence supports a hierarchical model in which aberrant fear processing in schizophrenia arises from disrupted salience attribution and impaired integration across cognitive, affective, and neurobiological levels. This intermediate dysfunction links molecular alterations to large-scale network disturbances and clinical symptom expression, providing a framework for more mechanism-based therapeutic strategies.

## 1. Introduction

Fear is an adaptive emotion elicited by imminent threat and serves as a coping mechanism crucial for survival [1,2]. However, defensive responses can become exaggerated or misdirected, leading to inappropriate reactions to neutral stimuli across multiple psychiatric disorders, including psychotic disorders. Fear is a phylogenetically conserved behaviors that links the perception of threat to coordinated defensive response such as fight-or-flight [3]. Its neurobiological implementation involves interactions between dopaminergic and glutamatergic systems, which are critically implicated in emotional processing [4] and to the regulation of fear responses [5,6]. In schizophrenia, dopamine dysregulation [7] has been associated with aberrant salience attribution [8], defined as the inappropriate assignment of significance to otherwise neutral stimuli, which may contribute to distorted environmental interpretations and fear-related experiences [9].

Neuroimaging studies of emotion recognition and facial expression processing in individuals with psychosis suggest that aberrant salience is associated with altered threat evaluation, including the misattribution of neutral facial expressions as threatening and abnormal attentional allocation to socially salient cues [10,11,12]. Positron emission tomography (PET) and functional magnetic resonance imaging (fMRI) studies further indicated that dopamine release correlates with conditioned fear responses [13] and is linked to amygdala activity during fear learning [14]. In schizophrenia, aberrant fear processing has been associated with reduced activation of limbic structures and an increase in medial prefrontal (mPFC) engagement during emotional processing tasks [15]. Converging evidence from preclinical and clinical studies suggests that dysregulated fear processing may represent a mechanistic bridge linking stress-related emotional dysregulation, psychotic symptoms, and delusional experiences [16,17,18,19].

This narrative review aims to integrate evidence across levels of analysis, including neurotransmission, synaptic plasticity, large-scale neural circuits, and neuroimaging findings, to characterize the shared neurobiology of fear and psychosis. Specifically, we address: (1) fear processing in relation to psychotic experiences and animal models of psychosis; (2) synaptic and molecular mechanisms of fear conditioning (FC) relevant to schizophrenia; (3) neuroimaging evidence of structural and functional network alterations associated with aberrant fear; and (4) the impact of pharmacological and non-pharmacological interventions on fear modulation.

## 2. Literature Search Strategy

Although this is a narrative review, the literature search was conducted in a rigorous and structured manner. Specifically, searches were performed in EMBASE, Scopus, and PubMed on 1 April 2026 using the following keyword combinations: (fear) AND (schizo*) AND (antipsychotic*); (fear) AND (schizo*) AND (dopamine*); (fear) AND (schizo*) AND (glutamate*); (fear) AND (schizo*) AND (GABA*); (fear) AND (schizo*) AND (serotonin*); (fear) AND (schizo*) AND (synap*); (fear) AND (schizo*) AND (PSD); and (fear) AND (schizo*) AND (salience*). Eligible studies included English-language articles published in peer-reviewed journals, with no temporal restrictions applied. Both original research articles and reviews were considered, whereas conference abstracts and commentaries were excluded.

## 3. Results

### 3.1. Fear Paradigms Used to Assess Behavioral Deficits in Rodent Models of Schizophrenia

#### 3.1.1. Fear Conditioning

FC comprises a set of Pavlovian associative learning paradigms at the interface of memory and emotion [3,20]. In FC, a subject learns to associate a neutral conditional stimulus (CS) with an aversive unconditional stimulus (US) such that, after repeated pairings, the CS alone elicits a conditioned fear response (CR) [21,22,23]. FC is implemented in several variants, notably cued FC (e.g., tone or light cues) and contextual FC (where the CS is the environment in which the US occurs, such as a conditioning chamber) [3,24] (Table 1). Cued FC is often used to model the behavioral response to immediate threats, whereas contextual FC models responses to more distal or environment-linked threats [25], and has been associated with functional disability [26]. These distinctions likely reflect the engagement of different neural circuits: cued FC primarily depends on the extended amygdala complex, while contextual FC relies heavily on the anterior cingulate cortex (ACC) [27] and hippocampus [28].

Trace fear conditioning (tFC) is a variant in which the CS and US are separated by a stimulus-free interval [29]. In situ hybridization and related approaches indicate that tFC specifically involves the ACC [30], a region involved in attentional processes across mammalians, including humans [31,32]. Preclinical studies have demonstrated roles for dopaminergic and glutamatergic neurotransmission in FC, acting in brain regions such as the basolateral amygdala and ventral tegmental area. Additionally, dopamine D1 and D2 receptors (D1R and D2R), as well as glutamate N-Methyl-D-aspartic acid (NMDA) and α-amino-3-hydroxy-5-methyl-4-isoxazplepropionic acid (AMPA) receptors (NMDAR and AMPAR), are required for fear learning and conditioning processes [33,34]. Together, these paradigms have provided mechanistic insights into fear acquisition and extinction and their relevance to psychiatric disorders [33,34].

Overall, FC paradigms indicate that associative fear learning relies on coordinated amygdala–hippocampal–prefrontal circuits modulated by dopaminergic and glutamatergic transmission, providing a mechanistic framework for studying threat learning relevant to psychiatric disorders.

#### 3.1.2. Fear Conditioning and Schizophrenia

Fear-related behavioral paradigms have been widely used to model emotional and cognitive alterations relevant to schizophrenia at the preclinical level [6]. At the molecular level, pharmacological models base on NMDAR antagonism have provided converging evidence for the involvement of glutamatergic dysfunction in FC alterations. Administration of ketamine has been shown to disrupt conditioned fear responses, including reduced freezing behavior, alongside increased locomotor activity in rodents, indicating a broad disruption of associative and affective processing [5,6,35,36]. The spontaneous hypertensive rats (SHRs) model has been employed to study deficits in emotional processing and their pharmacological reversal by antipsychotics [36,37]. In experimental paradigms, immobility or freezing represents the most widely used behavioral index of FC in rodents [36,37]. Multiple studies report reduced context-FC-induced freezing in SHR. Both ketamine and amphetamine decreased freezing in SHR and normotensive control rats, whereas treatment with antipsychotics such as haloperidol, risperidone, amisulpride, and ziprasidone can restore freezing deficits [36]. Notably, haloperidol has been reported to reduce freezing behaviors in some settings, while second-generation antipsychotics (SGAs) tend to increase freezing [36]. Neural selective GPCR-kinase interacting protein 1 (GIT1) knockout (KO) mice display deficits in FC and memory, together with reduced dendritic spine density in cortical neurons, relevant to schizophrenia neurobiology [38]. Similarly, β-neurexin (NRXN-β) KO mice showed impaired contextual fear memory [39]. Synaptic plasticity in the medial geniculate nucleus has been linked to increased freezing to auditory cues and to altered expression of AMPARs and synaptic scaffold proteins, including (SH3) and Multiple Ankyrin Repeat Domains 3 (SHANK) family members [40]. Neuroligin-2 R215H knock-in mice exhibit abnormal fear-related behaviors and disrupted γ-aminobutyric acid (GABA)ergic synaptic transmission in the mPFC [41]. Recent studies has also implicated Transcription Factor 4 (TCF4), a schizophrenia candidate gene [42], in FC deficits: transgenic mice overexpressing TCF4 showed impaired contextual and trace-aversive memory formation and concomitant deficits in prepulse inhibition (PPI) [43]. TCF4-overexpressing mice also showed deficits in both recent and remote trace fear-learning processes and a reduced ability to anticipate aversive events. These learning deficits depend on the ACC and hippocampus, as demonstrated by a decreased *c-Fos* expression in these regions [32]. *c-Fos* induction after FC training normally marks the activation of several emotion-related nuclei, including the *locus coeruleus*, paraventricular nucleus, basal lateral amygdala (BLA), *nucleus accumbens*, and ACC. Ketamine pretreatment prior to FC markedly attenuates gene induction across these regions except for the *nucleus accumbens*, whereas clozapine—but not haloperidol—blocks ketamine’s effects on gene expression and restores amygdala activity [5,6,35,36]. Neuregulin 1 (NRG1), a synaptic protein encoded by a gene linked to schizophrenia susceptibility [42], influences anxiety-related behavior in rodents through regulation of cued FC [44]. Although prefrontal cortex (PFC) dysfunction and NMDAR hypofunction are central to schizophrenia models, the PFC, amygdala, and hippocampus do not act in isolation; rather, they operate as an interacting network that collectively modulates fear-related memories and emotional learning [45,46,47].

#### 3.1.3. Fear Extinction

Fear extinction (FE) is a cognitive–behavioral process in which repeated presentation of the CS in the absence of the aversive US leads to attenuation or loss of the CF response [48,49]. FE may reflect the formation of new safety memories that coexist with, rather than erase, the original danger memory, since both danger and safety memories can be independently retrieved [50,51,52]. Retrieval of either memory is strongly context dependent [53,54]. Aberrant extinction has been implicated in the pathophysiology of anxiety disorders and post-traumatic stress disorder (PTSD), and the restoration of extinction processes is considered a mechanistic basis for many behavioral and cognitive therapies [49]. FE is also relevant to psychosis [55]: schizophrenia is characterized by impaired emotional processing, exaggerated neural responses to stimuli of low objective salience, and elevated arousal [55]. From the perspective of aberrant salience theory [56], the pathological attribution of significance to neutral stimuli may underlie the formation and persistence of delusional beliefs, a process that could be exacerbated by deficient FE or threat associations. Taken together, FE reflects a context-dependent inhibitory learning process that depends on prefrontal–amygdala–hippocampal interactions and NMDAR-dependent plasticity, and its disruption is implicated in both affective and psychotic symptom domains.

#### 3.1.4. Fear Extinction and Schizophrenia

Context-dependent recall of the extinction memory is impaired in schizophrenia patients [55]. An fMRI study demonstrated failure of extinction memory retrieval in schizophrenia that correlated with dysfunction of the ventromedial prefrontal cortex (vmPFC) [57], suggesting that FE retrieval may represent an interface between cognitive and emotional dysfunction in this disorder [57]. Converging evidence from preclinical models indicated that NMDAR antagonists disrupted FE in paradigms relevant to schizophrenia [58,59,60]. NMDAR antagonists infused into the hippocampus, BLA, and vmPFC impaired the extinction of both inhibitory-avoidance and context-FC [61]. Another report found the reversible inactivation of the BLA with 5-methyl-10,11-dihydro-5H-dibenzo[a,d]cyclohepten-5,10-imine maleate (MK-801), modulating both acquisition and extinction of fear to CS [62]. Animal models have also indicated that ketamine can impair the extinction of contextual threat-associated memory, possibly via dysregulation of the mammalian Target of Rapamycin complex 1 (mTORC1) signaling pathways [63,64]. In a genetic schizophrenia model [65], reelin haploinsufficient mice displayed an intact response to cued FC but delayed FE and impaired latent inhibition (LI) responses [66].

#### 3.1.5. Latent Inhibition

LI is a behavioral paradigm in which repeated pre-exposure to CS without pairing with a US reduces or delays subsequent FC. LI could be considered a moderator and attenuator of fear acquisition and expression, reflecting an organism’s ability to update or switch the associative value of a stimulus; both abnormally persistent and disrupted LI have pathological relevance. Several studies have demonstrated that dopamine modulates LI since dopamine agonists were shown to impair LI, whereas some antipsychotics (such as haloperidol and clozapine) were able to restore it [67,68,69]. The LI paradigm is widely used in animal models of schizophrenia because it indexes the capacity to ignore irrelevant stimuli, a process conceptually linked to aberrant salience attribution [70]. Like other psychosis-relevant paradigms (e.g., prepulse inhibition, PPI), LI is sensitive to dopaminergic manipulations and thus models aspects of attentional filtering and salience processing implicated in psychosis [71].

#### 3.1.6. Latent Inhibition and Schizophrenia

LI appears to be disrupted in acutely psychotic patients, but not in chronic schizophrenia patients [72]. LI is even closely associated with cognitive functioning, as it reflects the ability to filter out and ignore irrelevant stimuli, a process that is frequently disrupted in schizophrenia [73,74,75]. Multiple pieces of evidence showed the pharmacological modulation of LI in animal models of schizophrenia, reporting its disruption by amphetamine and its restoration by SGA in amphetamine-treated rats [76,77]. Furthermore, microdialysis studies have implicated both limbic structures and the mesolimbic–dopaminergic pathway in the neurobiological mechanisms underlying LI, highlighting the central role of dopamine-mediated salience processing in this phenomenon [67,78,79,80,81,82]. Converging evidence indicates that alterations in latent inhibition in schizophrenia and related animal models support the role of dopaminergic dysregulation in impaired attentional filtering and salience attribution, linking basic learning processes to cognitive and psychotic symptoms. To move from behavioral phenotypes to mechanistic understanding, it is necessary to consider how fear-related alterations observed in rodent models of schizophrenia emerge from synaptic and plasticity changes within key neural circuits.

### 3.2. Fear Experience and Synaptic Modulation: Implications for Schizophrenia

#### 3.2.1. Synaptic Mechanisms Underlying Fear Experience and Fear Memory

Preclinical evidence indicates that fear experiences can modulate synaptic function, leading to changes in protein expression, intracellular signaling pathways, and synaptic plasticity. Mouse models carrying the 22q11.2 deletion exhibit impaired synaptic transmission in thalamo-amygdala projections, resulting in deficit in emotional fear memory [83]. Similarly, Neuroligin-2 (Nlgn 2) Arg215 → His215 mutation knock-in (NL2 R215H KI) mice display reduced GABAergic neurotransmission within the mPFC, which contributes to abnormal fear responses [41]. Alterations in glutamatergic signaling have also been implicated in fear-related behavioral deficits. Glutaminase-KO mice exhibited behavioral and neurochemical abnormalities reminiscent of schizophrenia-like behavior, including disrupted glutamatergic synaptic function and impaired hippocampus-dependent context-FC [84]. Likewise, reduced expression of dysbindin-1 has been shown to impair NMDAR-dependent synaptic plasticity and context-FC [85]. Synaptic plasticity, particularly long-term potentiation (LTP) and long-term depression (LTD), are fundamental to the formation, storage, and modification of fear memories. LTP and LTD exert a critical function in the acquisition, retrieval, and extinction of CF and are differentially regulated by NMDAR subunits (GluN2A and GluN2B) [86,87]. Specifically, GluN2A-dependent signaling and the induction of LTP appear to facilitate the acquisition and stabilization of a learned fear response, whereas GluN2B-dependent signaling and LTD are thought to promote fear extinction by weakening previously established fear associations [86].

Disruption of these processes across genetic models relevant to schizophrenia impairs fear learning and extinction, highlighting synaptic plasticity as a key convergence point between fear circuitry and psychosis-related dysfunction.

#### 3.2.2. PSD Proteins Relevant to Schizophrenia Involved in Fear-Related Behaviors

**Figure 1:**
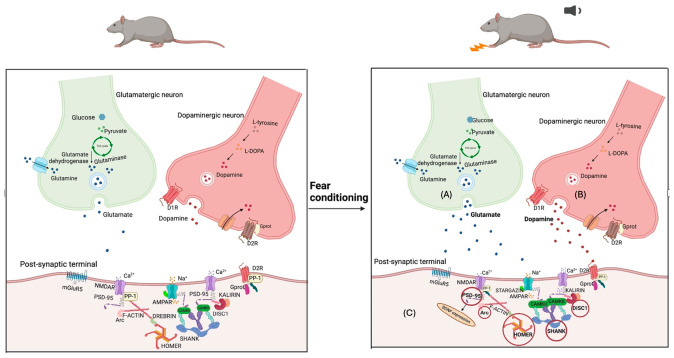
Conditioned fear stimuli (exposure to tone and footshock) regulate (A) the synaptic dopamine release through dopamine receptor activity, (B) the glutamatergic neurotransmission via NMDAR, and (C) the postsynaptic density protein expression. Abbreviations: Arc, activity-regulated cytoskeleton-associated protein; AMPAR, α-amino-3-hydroxy-5-methyl-4-isoxazolepropionic acid receptor; BDNF, brain-derived neurotrophic factor; Ca^2+^, calcium; CAMKII, calcium/calmodulin-dependent kinase II; cAMP, cyclic adenosine monophosphate; D1R, dopamine D1 receptor; D2R, dopamine D2 receptor; DISC1, disrupted in schizophrenia 1; GKAP, guanylate kinase-associated protein; GSK3, glycogen synthase kinase 3; Gprot, guanine nucleotide-binding proteins; L-DOPA, l-3,4-dihydroxyphenylalanine; mGluR, metabotropic glutamate receptor; Na^+^, sodium; NMDA, N-methyl-D-aspartate; PP1, protein phosphatase 1; PDE4, phosphodiesterase-4; PSD-95, postsynaptic density-95; SHANK, SRC homology 3 domain and multiple ankyrin repeat domains; TCA, tricarboxylic acid cycle. Created with BioRender.com de Bartolomeis (2026).

Postsynaptic density (PSD) is an electron-dense specialization of excitatory glutamatergic synapses that comprises a highly organized protein network, including ionotropic and metabotropic glutamate receptors, cell-adhesion molecules, and cytoskeletal and scaffolding proteins, as well as intracellular signaling components. Electron microscopy studies have revealed that both FC and conditioned inhibition are associated with dynamic structural remodeling of the postsynaptic compartment, including glutamate receptor trafficking and alterations in PSD architecture [88,89,90]. Notably, several studies have reported PSD enlargement following FC, a phenomenon associated with increased polyribosome accumulation within the dendritic spine of the lateral amygdala. These findings suggest that fear learning induces de novo local protein synthesis and dendritic translation, processes that are critical for the consolidation of fear memories [89]. Table 2 and Figure 1 summarize the most relevant evidence regarding PSD proteins, PSD-associated/synaptic signaling protein, and their putative roles in fear processing.

Accumulating evidence further indicates that fear memory formation and fear extinction are regulated by epigenetic mechanisms that influence the expression and function of PSD-associated proteins. These molecular modifications contribute to activity-dependent synaptic remodeling and represent context-dependent molecular signatures of FC fear extinction, and fear-related associative learning [91,92,93,94]. Overall, evidence indicates that FC induces dynamic remodeling of the PS, involving structural reorganization, receptor trafficking, and activity-dependent local protein synthesis. These processes are further modulated by epigenetic mechanisms that regulate PSD-associated gene expression, supporting a role for synaptic structural plasticity in linking fear learning, memory consolidation, and schizophrenia-related synaptic vulnerability.

**Table 2:** Findings on the PSD proteins, PSD-associated/synaptic signaling protein, and their putative role in fear processing. Abbreviations: CA1, Cornus Ammonis-1; nNOS, Neuronal nitric oxide synthase; NMDAR, N-methyl-D-aspartate receptor; BLA, basolateral amygdala; BDNF, brain-derived neurotrophic factor; CS, conditioned stimulus; DISC1, disrupted in schizophrenia 1; ErbB4, Erb-B2 receptor tyrosine kinase 4; FC, fear conditioning; HPA, hypothalamic–pituitary–adrenal; KD, knockdown; KO, knockout; NRG, neuregulin; nNOS, neuronal nitric oxide synthase; PSD-95, postsynaptic density-95; PPI, prepulse inhibition; Shank, SRC homology 3 domain and multiple ankyrin repeat domains; AMPAR, α-amino-3-hydroxy-5-methyl-4-isoxazole propionic acid receptor; Disc1-KD; Disrupted-in-Schizophrenia 1 Knockdown.

PSD Proteins and PSD-Associated Proteins	Main Outcomes	References
PSD-95	The ligand-binding-deficient PSD-95 affects anxiety-like behavior in FC tests. Two distinct PSD-95 domains have been associated with fear memory: the guanylate kinase domain involved in the aversive memory process and the domain relevant for nNOS, inducing an increase in BDNF signaling. In vivo–ex vivo electron microscopy and an electrophysiology study in mice showed CA1 hippocampal phosphorylation of PSD-95 at Serine 73, suggesting a possible molecular mechanism required for updating contextual fear memory. Recent experimental evidence indicates that disrupting the Src-PSD-95 interaction enhances NMDAR signaling and rescues trace fear conditioning deficits in a schizophrenia model.	[95,96,97]
NRG1	Type II NRG1 in rats leads to abnormal responses to the cued-FC paradigm and exposure to environmental stressors during adolescence has been reported to affect fear responses during adulthood, shaping adult behavior and HPA axis neurocircuitry function. The NRG1-ErbB4 pathway restores deficits in long-term contextual fear memories via ErbB inhibitors. Impairment in contextual fear memories, as well as in social recognition memory and sensorimotor gating, has also been observed in transgenic male mice that mimic the overexpression of NRG1 type III. The type III NRG1^+/−^ male mice from mutant fathers, but not mothers, showed deficits in contextual fear-associated memory. Altered NRG1 signaling during adolescence or adulthood contributes to sharing specific behavioral phenotypes, including impaired FC, hyperlocomotion, and impaired working memory. It has also been demonstrated that ErbB4 KO mice exhibit a specific set of behavioral phenotypes associated with schizophrenia, including memory in contextual fear learning and a trend for a deficit in sensorimotor gating. Mutations in NRG3 (a paralog of NRG1) are linked to cognitive and psychotic symptom severity in the NRG3 KO mouse model, exhibiting hyperactivity, impaired PPI, and altered FC.	[98,99,100,101,102,103,104,105]
Homer1a	Homer1a KO mice display impaired long-term contextual fear conditioning, albeit having intact short-term conditioning performance. A preclinical rat study demonstrated that two short forms of Homer1 are strongly modulated by context-FC in the hippocampus, resulting in consolidation, retrieval, and extinction of associative fear memory that, in turn, regulates Homer1a expression. Epigenetic studies have highlighted the prominent role of Homer1 in consolidating fear acquisition, showing that both in vitro and in vivo manipulations result in a reduction in Homer1a promoter H3K9 methylation in amygdala cells and an increase in Homer1a promoter H3 acetylation in hippocampal cells.	[106,107,108,109]
Arc	Arc protein expression in the lateral nucleus of the amygdala is relevant to restoring an auditory fear memory. Arc was found to be involved via AMPAR in the extinction of threat-associated memory, and Arc expression modulation in the BLA is relevant for the long-term extinction of FC. In a mouse model of auditory FC, Arc expression was evaluated after a CS. In this paradigm, there was an increase in Arc levels in the amygdala and the infralimbic PFC from 2 to 6 h after 20 trials of CS presentation, whereas in the BLA, it was 2 h after one trial of CS, probably due to different effects of Arc activation depending on neural circuits.	[110,111,112,113,114]
Shank	Shank1^−/−^ mice showed preserved cued-FC-dependent learning and memory of aversive stimuli, while context-FC memory resulted in a reduction. Shank3A^−/−^, Shank3A^+/−^, and Shank3B^−/−^ mice exhibited intact cued and contextual FC-induced learning and memory. Mice with Shank3Q321R/Q321R mutations do not show significant alterations in the acquisition or extinction of contextual fear memory.	[115,116,117,118]
DISC1	Transgenic mice have shown that manipulating DISC1 results in hyperactivity, increased immobility in the forced swim test, deficits in PPI, decreased sociality, impaired working memory, and FC. Disc1-KD in hippocampal astrocytes exhibit reduced cued-FC-dependent freezing without altering training or context-FC-dependent freezing, while Disc1-KD in cortical astrocytes had no impact on training, context, or cued-FC-dependent freezing.	[119,120,121,122]

### 3.3. Fear and Schizophrenia: Focus on Intracellular Signaling

An in vitro study demonstrated that Akt, a downstream effector inhibited by phosphoinositide 3-kinase (PI3-K) phosphorylation, blocks fear-associated memory traces formation, suggesting a potential role for this signaling cascade in FC [123]. Within the amygdala, protein kinase B (PKB/Akt) has been shown to regulate light-cued FC through a broader intracellular pathway involving brain-derived neurotrophic factor (BDNF), tropomyosin receptor kinase B (TrkB), mitochondrial adaptor p66 (Shc), PI3-K, Akt, and mitogen-activated protein kinase (MAPK) cascade [124]. Consistently, phosphorylated Akt (pAkt) levels are increased in the BLA of mice exhibiting high anxiety-related behavior (HAB) compared to normal anxiety-related behavior (NAB) mice following cued FC training [125]. HAB mice, characterized by a genetic predisposition to heightened anxiety, display enhanced fear memory acquisitions, together with increased expression of associated neurochemical markers.

Further mechanistic evidence shows that ß-catenin is upregulated, whereas phosphorylated calcium–calmodulin-dependent protein kinase II (pCaMKII) is decreased in the BLA of both trained HAB and NAB mice compared to non-trained mice. In this context, intrahippocampal administration of wortmannin, a PI3-K inhibitor, improved long-term context-FC memory in trained animals, potentially by facilitating consolidation processes [126]. In contrast, Akt knockout (Akt^−/−^) mice exhibited impairment in context-FC, spatial memory retrieval, and displayed a mild anxiety-like phenotype [127]. Finally, the PI3-K/Akt/m-TOR signaling pathway within the mPFC played a prominent role in tFC as pharmacological inhibition of PI3K or mTOR in the mPFC and disrupts long-term tFC memory in rodents [128]. In addition, the involvement of the PI3-K/Akt/MAPK pathway has been investigated in FE paradigms. In this context, FE has been shown to be enhanced by DCS, whereas intra-amygdaloid administration of PI3-K and MAPK inhibitors disrupts DCS-induced facilitation of FE [129]. Preclinical studies further indicated that long-term unpredictable stress decreased phosphorylation levels of mTOR and its downstream signaling components within the amygdala [130]. These alterations were accompanied by decreased phosphorylation of ERK-1/2, Akt-1, and AMPAR subunit glutamate receptor 1 (GluR1), while no significant changes were observed in other brain regions such as the frontal cortex, hippocampus, or dorsal raphe nucleus. This pattern suggests amygdala-specific molecular dysregulation, supporting the potential of mTOR-related pathways as pharmacological targets in stress-related psychiatric disorders [130]. Stress hormones are also key modulators of fear learning and emotional regulation through the hypothalamic–pituitary–adrenal (HPA) axis. Glucocorticoid signaling also plays a critical role in emotional processing. Experimental evidence shows that activation of glucocorticoid receptors can facilitate high-frequency network oscillations in the ACC, a region strongly implicated in fear regulation and cognitive control [130]. Finally, genetic factors influencing synaptic plasticity may further contribute to alterations in fear-related memory processes. For instance, conditional deletion of the voltage-dependent L-type calcium channel subunit α1C (CACNA1C) gene, encoding the CaV1.2 calcium channel in D1R-expressing neurons, produces significant impairments in aversive learning and spatial memory [131].

Collectively, these findings indicate that intracellular signaling cascades, including PI3K/Akt, MAPK, and mTOR pathways, converge on the regulation of synaptic plasticity mechanisms underlying fear learning and extinction. Dysregulation of these signaling networks, particularly within the amygdala and PFC, contributes to altered fear memory formation, stress sensitivity, and behavioral abnormalities relevant to schizophrenia. At a higher level of organization, these synaptic alterations are regulated by neurotransmitter systems involved in the modulation of fear-related neural circuits.

### 3.4. Neurotransmission of Fear Mechanisms in Schizophrenia Models

#### 3.4.1. Dopamine’s Role in Fear Processing

The role of dopamine in processing emotionally salient stimuli and memory is well established [131,132,133]. Dopamine affects fear acquisition and expression in several brain regions including the PFC, hippocampus, amygdala, and *nucleus accumbens* [20,134,135,136]. Specifically, increased dopamine in the *nucleus accumbens* has been associated with the occurrence of memories of aversive stimuli. This finding is especially relevant to the psychopathology of schizophrenia, since subcortical dopamine overflow is regarded as a biochemical hallmark of the disorder in a subset of patients, and it may be linked to alterations in fear memory [137,138]. Dopaminergic neurons in the ventral tegmental area (VTA) respond to fearful stimuli, impacting FC [139,140,141,142]. Moreover, dopamine levels peak in the amygdala, and the *nucleus accumbens* receives a dopamine release during stressful events, influencing fear response [143]. In dopamine-deficient mice, tested in the fear-potentiated startle (FPS) paradigm, the FPS response is abolished; L-Dopa administration, as well as tyrosine hydroxylase gene reintegration in the BLA and VTA, reverted the FPS deficit [33].

In methamphetamine-treated rats, conditioned fear stress elicited increased dopamine release [144]. The BLA—receiving cortical and subcortical inputs—serves as the primary sensory interface, whereas the CeA is the main output nucleus that mediates fear expression and fear memory extinction [20,145]. The lateral nucleus of the amygdala is an early site of integration for sensory information about both CS and US [146]. The indirect connections between the BLA and CeA are provided by intercalated paracapsular islands (IPCs), a group of GABAergic neurons that resemble the medium-sized spiny neurons (MSNs) of the striatum [147,148]. IPCs constitute the principal D1- and D2-receptor-expressing population within the amygdala and receive dopaminergic input from the ventral tegmental area [149]. In addition, electrophysiological studies have demonstrated long-term potentiation (LTP) induction in the BLA [150] and D1-receptor-mediated responses in the CeA [151]. Experimental studies have shown that social stress can recruit associative memory neurons within interconnected cortical circuits involving the medial prefrontal, auditory, and somatosensory cortices through D2R-dependent mechanisms, leading to both fear memory formation and schizophrenia-like behaviors [152].

PET and fMRI studies have clarified the role of D1R and D2R in humans, demonstrating their modulation of fear acquisition and retrieval. These studies offer therapeutic potential for conditions like schizophrenia [61,152,153,154,155,156,157,158].

Taken together, these findings indicate that dopaminergic signaling modulates fear acquisition and expression across cortico-limbic and striatal circuits, with D1R/D2R-dependent mechanisms contributing to both fear learning and salience attribution processes relevant to schizophrenia.

#### 3.4.2. Glutamatergic Modulation of Fear Responses and Fear-Related Memory

Studies in rats exposed to FC show glutamate increases in the mPFC and freezing behavior, mediated by the NMDAR and glycine modulator site [159]. FC induces neural functional changes in the lateral amygdala via Ca^2+^-dependent associative LTP [160,161,162]. PCP treatment in mice disrupts NMDAR-induced signaling [163], which is linked to schizophrenia-like symptoms [164]. *SynGAP* mutant mice exhibit FC deficits and emotional disturbances due to abnormal amygdala function related to NMDAR hypofunction [3,24,165]. NMDAR antagonists affect aversive memory and learning paradigms, impacting fear expression and extinction [166,167,168,169,170]. Multiple lines of evidence associate metabotropic glutamate receptor (mGluR) 1 and mGluR5, along with their coupled scaffolding proteins network, with both the behavioral and molecular signatures of fear induction and persistence of fear-like behavior, indicating a profound synaptic reorganization at glutamatergic postsynaptic density [171,172]. Experimental models have shown that genetic alterations of the mGluR5 can produce schizophrenia-like behavioral phenotypes and reward-related alterations in a sex-dependent manner [171,172]. Interestingly, antipsychotics commonly used in schizophrenia have been shown to significantly modulate the gene expression of multiple glutamatergic synaptic proteins, including the ones implicated in fear behavior, when administered alone or in augmentation with other psychotropic drugs [173,174,175,176,177,178]. Additionally, mGluR agonists and antagonists modulate fear responses, offering potential therapeutic targets [14,179,180]. Mutations in the mGlu7 receptor gene increase schizophrenia susceptibility, highlighting its role in fear regulation [181,182]. These findings underscore the complex interplay of glutamatergic transmission in fear processing and its implications for schizophrenia pathology.

#### 3.4.3. Other Neurotransmitter Implications in Fear and Schizophrenia

The GABAergic system is believed to play a role in the control of cognition and emotions, such as fear, in schizophrenia. Aberrant GABAergic transmission in the mPFC disrupts fear responses and synaptic modulation in animal models [41]. The amygdala’s modulation of sensorimotor gating and fear conditioning via GABAergic interneurons has a role in fear regulation [183,184]. Reduced GABA turnover in the cortex, hippocampus, and striatum leads to abnormal fear responses in schizophrenia-like mice [185]. Hyperactivity in the mPFC to neutral stimuli correlates with deficits in LI and aberrant salience in schizophrenia [10,70,186,187,188]. Blocking GABAergic transmission in the mPFC increases the phasic activity of midbrain dopaminergic neurons, impacting LI. Dysregulated GABAergic neurotransmission in the PFC contributes to disturbances in LI and fear learning in schizophrenia [189,190]. Increased transmembrane neural cell adhesion molecule (NCAM) expression alters synaptic connectivity of GABAergic interneurons in fear-conditioned mice. Modulation of nicotinic receptor α7-nAChRs influences GABAergic inhibition in the BLA, relevant to schizophrenia, with potential therapeutic implications [191,192,193,194,195,196,197,198,199,200]. Abnormal 5-hydroxytryptamine (5-HT) activity, notably through 5-HT2A receptor activation, influences fear extinction and neurotransmission in the *nucleus accumbens*, suggesting avenues for therapeutic intervention [201,202,203,204]. Neurotransmission and molecular mechanisms in fear-conditioned animal models are summarized in Table 3 and Figure 1.

Overall, these data indicate that GABAergic and serotonergic systems contribute to the fine-tuning of fear responses through inhibitory control of cortico-limbic circuits and modulation of extinction and salience filtering processes. Dysregulation of these systems, together with dopaminergic and glutamatergic abnormalities, suggests a broader imbalance in excitatory–inhibitory control underlying fear-related dysfunctions in schizophrenia. While studies in schizophrenia models have highlighted the role of neurotransmitter systems in fear processing, converging evidence from brain imaging studies in patients has demonstrated that these neurochemical alterations are associated with dysfunctions in corticolimbic fear circuits.

### 3.5. Brain Imaging Studies and Aberrant Fear in Schizophrenia Patients

#### 3.5.1. Aberrant Fear in Schizophrenia and Brain Networks

Preclinical evidence identifies specific brain circuits involved in fear, while human studies report mixed results [212]. Neuroimaging studies [213] suggest that the neurobiological basis of fear might correspond to brain regions involved in arousal, reward, and punishment [214,215] (Table 4). Clinical evidence is focused on the amygdala, which is involved in the recognition [216], expression [217], and experience of fear [218]. In this neurofunctional framework, neuroimaging studies, through the identification of foundational regions of interest (ROIs) involved in fear processing, showed a significant role of the mesolimbic network consisting of the ventral striatum, ventral and medial frontal areas, and anterior and middle cingulate cortex, as well as the amygdala and parahippocampal cortex [219,220]. In this context, the paradigm of an anxiety-induction PET study in normal subjects demonstrated a regional cerebral blood flow (rCBF) increase in the ACC and the claustrum–insular–amygdala regions associated with induced anxiety, whereas the increase in the orbitofrontal cortex was associated with anticipatory anxiety [221,222]. Furthermore, fear could be processed by multiple brain networks, where hubs such as the amygdala could represent nodes of a more extended set of structures [223]. The formation of amygdala-dependent fear memories in both rodents and humans appears to be enhanced by endogenous dopamine release, supporting an evolutionarily conserved neurochemical mechanism related to fear [13,224]. fMRI studies in humans have more consistently observed increased blood-oxygenation-level-dependent (BOLD) levels during these processes in the association cortices [225]. FC in humans is associated with a pattern of neural activation in a putative network of brain regions that mirror the anatomical areas of the ‘central autonomic–interceptive network’ (where the ACC and anterior insular cortex are represented as the main input–output nodes) pointing to the neural substrate of conscious fear processing [225]. Growing evidence suggests that FC in humans may activate a set of brain regions, consistent with an extended ‘fear network’ [226,227,228]. Specifically, the connection between the amygdala and other brain regions enables the processing of salient stimuli [229]. In this frame, the anterior insular cortex would mediate higher-level appraisal and anticipatory processes relevant to the conscious experience of fear [230]. Human FC-fMRI studies have demonstrated an evoked central autonomic–interoceptive network response that represents the threat appraisal and response processes involving cognitive, motivational, and psychomotor domains [225]. Interestingly, fMRI studies on the generalization of FC in healthy subjects have shown that the salience network (SN) (including the anterior insula and dorsomedial frontal cortex) and the default network (DN) (including the frontal and middle parietal cortices and the lateral parietal and temporal cortices) show opposite response patterns with statistically significant activation in the SN and deactivation in the DN [231,232,233]. Confluent lines of evidence suggest that the amygdala is involved in the acquisition, retention, and expression of FC memory [227]. Functional neuroimaging studies have confirmed the subcortical and cortical structures involved in FC paradigms, revealing the role of the medial temporal lobe in modulating associative changes in the cortex during fear-related processes. It has been hypothesized that the amygdala mediates between brain systems by reflecting the changes in synaptic connections implicated in aberrant fear-related processes at the cortical level [227,234]. Collectively, neuroimaging evidence suggests that fear processing is supported by distributed cortico-limbic and large-scale functional networks, including salience and default-mode systems, with the amygdala acting as a central hub integrating emotional, interoceptive, and cognitive signals. Dysregulation of these networks in schizophrenia contributes to impaired threat evaluation and aberrant salience processing.

#### 3.5.2. Aberrant Fear and Connectivity in Clinical Studies

Multiple lines of evidence suggest that disruptions in both pleasant [235,236] and aversive [55,57,186,237] associative learning and memory processes in schizophrenia may lead to downstream changes in cognitive functions and higher-order behaviors, following a “bottom-up” model representative of the functional impairment in schizophrenia [11]. In this context, Tuominen and coworkers showed an abnormality in the generalization of FC responses in individuals with schizophrenia [238]. Furthermore, a trend towards decreased SN activation during FC in schizophrenia suggests possible abnormalities in fear learning related to positive symptoms treated with antipsychotics in schizophrenia patients [238]. In addition to lower activation of the SN, reduced deactivation of the DN during fear generalization in schizophrenia patients has also been found [239,240,241]. The pattern of DN activity anticorrelates with the SN one, pointing out that the dysregulation of this crosstalk could potentially challenge brain homeostasis [242]. Clinical evidence in schizophrenia has found decreased connectivity between the SN and DN [243,244], as well decreased structural integrity [243,245] and centrality [246]. Preclinical evidence demonstrated that the activation of NMDAR in the rat prelimbic cortex during the consolidation or recovery phases of FC-induced fear generalization [247] raises these receptors as a promising therapeutic target. Considering that the gradient of fear generalization is shaped by a dynamic interaction between excitatory and inhibitory inputs [248], which appears to be imbalanced in schizophrenia, it has been suggested as a mechanism underlying aberrant fear to be investigated as a further therapeutic target in schizophrenia [249,250]. Aberrant fear could result from the negative emotional valence assigned to auditory verbal hallucinations (AVHs) in patients with schizophrenia, which is considered a predictor of disease severity [251]. Magnetic resonance spectroscopy (MRS) studies found that the negative emotional valence of AVHs in frontal and temporal brain regions correlated with lower levels of glutamate + glutamine (Glx) [251]. Specifically, expressions of emotional resistance, such as fear, rather than behavioral resistance, were found to be a predictor of reduced Glx [251]. The contribution elicited by the Glx reduction in ACC could be related to the direct impaired cognitive control of AVHs and indirect emotional dysregulation via inhibitory signals to the amygdala [252], possibly related to schizophrenia patients’ experiences of emotional resistance, including aberrant fear.

#### 3.5.3. Aberrant Fear and Amygdala Abnormalities in Schizophrenia

**Figure 2:**
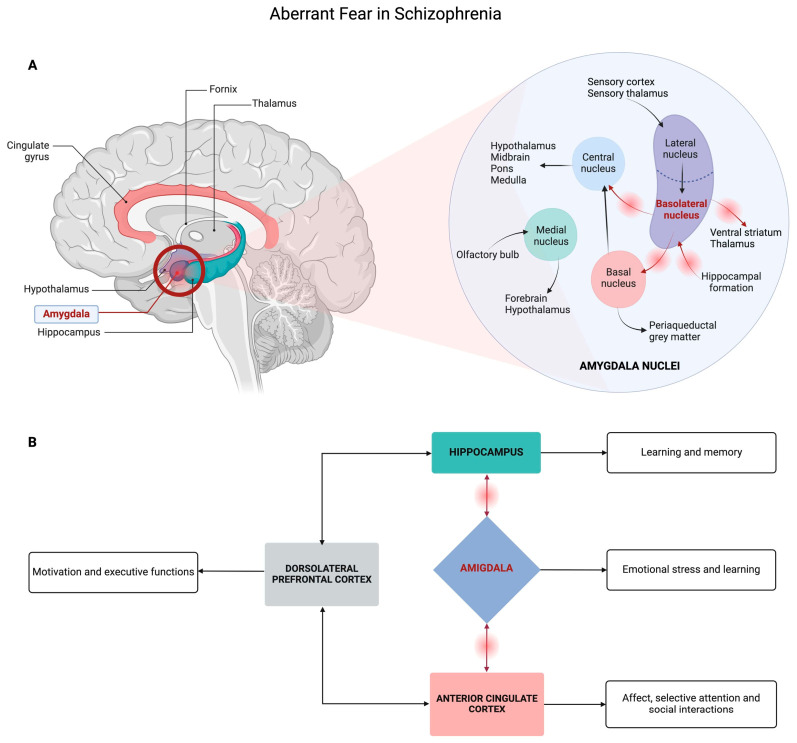
Aberrant fear network in schizophrenia. (**A**) The corticolimbic system is composed of several brain regions, including the anterior cingulate cortex, hippocampal formation, and basolateral amygdala. The anterior cingulate cortex plays a key role in the conscious processing of emotions and selective attention responses. Emotional learning is mediated through interactions between the basolateral amygdala and hippocampal formation, and motivational responses are processed via the dorsolateral prefrontal cortex. (**B**) Schematic representation of the amygdala’s central role in aberrant fear effects on cognition and processing mode in schizophrenia. Created with BioRender.com de Bartolomeis (2026).

The amygdala has been implicated in the abnormal emotional processing observed in patients with schizophrenia, as well as in alterations in fear-conditional avoidance learning [253,254,255]. Specifically, the amygdala appears to be involved in processing the emotional meaning of single stimuli and complex situations [3,256], in the perception of emotions [257,258], in social cognition [216,259,260], and in the ability to attribute emotional mental states to others, possessing a functional theory of mind [261]. In addition to its involvement, other regions also play a role in the functioning of the emotional brain, such as the mPFC, orbitofrontal cortex, ACC, and insula [262,263,264,265]. The BLA plays a central role, interacting with a frontotemporal system linking several components of the corticolimbic system, including the ACC and the hippocampal formation [266] (Figure 2). The ACC cooperates with the BLA in mediating conditioned memory [267]. Although the amygdala is probably not the site of long-term storage of declarative memory, it appears to influence memory recall mechanisms in other brain regions, such as hippocampal formation [268,269]. The BLA could contribute to the neural plasticity associated with encoding FC [270] and goal-directed behavior [271], assuming a function in aberrant fear in schizophrenia (Figure 2). An intriguing model proposed that the lateral amygdala may act as a specific sensory interface for simple unimodal stimuli, while the BLA may serve as a sensory interface for complex stimuli, connecting the motor circuits of the striatum and cortex to elicit responses to emotional stimuli. Furthermore, this model also included the CeA, which can activate brainstem areas involved in controlling the involuntary aspects of emotional reactions (e.g., autonomic responses) [256]. Reduced prefrontal connectivity may lead to emotional response reduction (e.g., emotional blunting), explaining the negative symptoms of schizophrenia. On the other hand, reduced prefrontal control of the BLA may lead to aberrant CeA function, which generates increased involuntary emotional reactivity (e.g., hyperarousal) [272] and the subjective experience of fear [192]. In this context, the influence exerted by the CeA and BLA on the mesocorticolimbic dopaminergic system has been suggested [273]. Specifically, the CeA controls tonic dopaminergic activity in the VTA via an indirect pathway, whereas the BLA evokes transient increases in dopamine efflux in the *nucleus accumbens* and PFC. Increased tonic dopamine levels may be related to reduced inhibitory control of the CeA, whereas reduced phasic dopamine release may be related to BLA lesions. This model is consistent with [15O] water PET findings on the increase in amygdala and ventral striatum tonic rCBF in patients with schizophrenia [274] and phasic alterations in response to emotionally salient stimuli [275], with the dysregulated contribution of the dopaminergic system in processing emotion perception [4]. Furthermore, it has been argued that reduced amygdala reactivity in patients with schizophrenia to negative facial stimuli coincides with an alteration in functional coupling between the amygdala and the subgenual cingulate cortex, a subdivision of the ACC [276]. Preclinical studies have shown that ablation of the amygdala results in the loss of control over fear responses [277], while clinical studies associate amygdala damage with a lack of conditioned fear response and a poor ability to assign affective salience to emotional stimuli [278]. Aberrant fear may, therefore, be correlated with a lesion of the amygdala combined with reduced interconnectivity with the PFC within reciprocal negative emotional feedback between reduced emotional expression (affective flattening) and defective emotion recognition [192] (Figure 2).

#### 3.5.4. Neuroimaging Studies on Amygdala Functional and Structural Changes in Schizophrenia

Meta-analytical evidence from structural magnetic resonance imaging established a bilateral amygdala volume reduction of 6–10% in patients with schizophrenia [279,280,281]. In addition, studies showed that the left amygdala may be more affected than the right in this disorder [282]. The reduction in gray matter density of the amygdala was associated with aging in patients with schizophrenia, suggesting that disease duration contributed to functional changes in the amygdala. Conversely, another MRI meta-analysis on the dimensions of various brain structures in patients with first-episode schizophrenia found that the right and left hippocampus, but not the amygdala, showed a significant volume reduction [283]. These results indicate a primary pathophysiological change affecting the left hippocampus in patients experiencing first-episode schizophrenia, confirming that amygdala modifications may be secondary to the onset of the disease [284]. Other neuroimaging evidence in children and adolescents with schizophrenia has reported a decrease in volume in both these regions and the ACC, underlining the progressive neurodevelopmental nature of schizophrenia [285]. A [15O] water PET study using the International Affective Picture System (IAPS) [286] examined limbic–emotional function in schizophrenia, showing hypoactivity of the right amygdala in patients compared to healthy controls. On the other hand, the study reported that left amygdala hyperactivity correlated with positive symptoms of schizophrenia [275]. In line with these findings, Taylor and coworkers suggested that reduced rCBF in the amygdala might be related to defects in emotion recognition, and increased rCBF might be associated with positive symptoms, consistent with a general failure to process salient stimuli in schizophrenia [275]. In a PET study using the IAPS, patients with schizophrenia exhibited decreased phasic neural responses in the right ventral striatum to salient stimuli and increased basal tonic responses in both the right ventral striatum and bilateral amygdala, as observed via rCBF measurements [274]. A [15O] water PET study with drug-free schizophrenia patients, for at least three weeks, and healthy controls showed that the patients did not exhibit amygdala rCBF increase during the visual image tasks [287]. Despite preclinical studies demonstrating an antipsychotic impact on amygdala function [288], neuroimaging findings on treated schizophrenia patients have not found correlations between medication dosage and amygdala activation [289,290]. In conclusion, structural and functional neuroimaging studies indicate that schizophrenia is associated with stage-dependent alterations in amygdala and hippocampal integrity, together with abnormal functional activation patterns during emotional processing. These changes are consistent with disrupted limbic responsivity and impaired salience attribution mechanisms. The identification of aberrant fear processing and disrupted corticolimbic activity in schizophrenia patients through brain imaging studies offers a mechanistic basis for investigating how antipsychotic drugs may influence these neural systems.

### 3.6. Antipsychotic Drugs and Fear: Implications for Schizophrenia

Clinical and preclinical evidence on antipsychotic drugs in fear and schizophrenia studies is reported in Table 5.

#### 3.6.1. Preclinical Evidence and Translational Issues for the Clinics

Preclinical evidence indicates that antipsychotic drugs modulate fear-related behaviors through convergent effects on dopaminergic, serotonergic, and GABAergic signaling within corticolimbic circuits. Importantly, these findings suggest that fear-conditioning paradigms represent a translational bridge between molecular mechanisms and emotional dysfunction observed in schizophrenia. An in vivo microdialysis study of methamphetamine-sensitized and fear-conditioned rats examined differences in the effects of aripiprazole and haloperidol on dopamine release. After exposure to a CS, methamphetamine-sensitized rats showed significantly higher dopamine release in the amygdala compared to non-sensitized rats. Aripiprazole suppressed the increase in both tonic and phasic dopamine levels in the amygdala of fear-conditioned rats, while haloperidol only decreased phasic release and increased the basal level of extracellular dopamine in the amygdala [300]. These differences suggest that distinct pharmacodynamic profiles may translate into differential modulation of salience processing and emotional reactivity. Consistent with this, multiple studies demonstrate that antipsychotic compounds differentially affect conditioned freezing and avoidance behaviors. A preclinical study in rats investigated the effects of antipsychotic drugs on fear-induced conditioned freezing (determined through foot shock stress) in which the atypical agent clozapine decreased the acquisition of conditioned freezing via a dose-related mechanism. Other compounds, such as olanzapine, haloperidol, spiperone, nemonapride, and raclopride, also inhibited conditioned freezing. Furthermore, inhibition of conditioned freezing correlated significantly with Ki values for dopaminergic receptors, suggesting a D4R-mediated mechanism on the modulation of conditioned freezing acquisition [298]. A study on fear models demonstrated that clozapine and chlordiazepoxide, but not haloperidol, improved shock conditioning. The authors showed significant differences in the effects of various antipsychotics on two categories of conditioned fear responses (active and reactive) [292]. Specifically, conditioned fear responses, such as freezing, environmental passive avoidance, increased body temperature, or the number of vocalizations, are innate, reflexive, and species-typical responses to threats, classified as “reactive conditioned fear responses.” In contrast, active avoidance of a fearful stimulus requires animals to make a voluntary and intentional motor action to the danger, considered a “conditioned active fear response” [292,305]. In this context, antipsychotics appear to have a beneficial effect primarily on the active avoidance response [306]. Haloperidol improves the active avoidance response but has a little or even enhanced effect on conditioned reactive fear, while clozapine and olanzapine have inhibitory effects on both types of fear responses [292]. Clozapine and olanzapine can dose-dependently increase allopregnanolone, a positive modulator of the GABA_A_ receptor, in the rat cerebral cortex and hippocampus [307,308,309], contributing to the beneficial effects on the stress response and conditioned fear regulation [292]. Preclinical evidence has shown that perospirone, a 5-hydroxytryptamine 2 receptor (5-HT2R) and D2R antagonist, as well as the other serotonergic and dopaminergic antagonists (SDAs), namely clozapine, risperidone, ritanserin, and ketanserin, are effective in ameliorating the freezing behavior induced by conditioned fear stress in a dose-dependent manner. Other studies have also supported the hypothesis that many antipsychotics (such as haloperidol and clozapine) have beneficial effects on PPI and FC [310,311,312]. Martin et al., in a study on rats after intracerebroventricular administration of excitotoxin, analyzed contextual and attended FC and reverse spatial learning (that were found to be altered). They then administered some antipsychotic drugs and evaluated [299] that after quetiapine administration, deficits in contextual and attended FC improved, while reverse learning did not improve, suggesting its procognitive effects in rats with hippocampal neuropathy [313]. In a preclinical microdialysis study, clozapine was proven to be superior to haloperidol in inhibiting excessive dopamine release (brought about by FC) in the amygdala of methamphetamine-sensitized rats [295]. Moreover, it was shown that rats conditioned to fear exhibited freezing behavior, reversed by some antipsychotics (such as asenapine and buspirone) [301]. Aripiprazole was reported to be effective in reducing freezing time in a preclinical study of context-FC [293]. Lurasidone may improve emotional control in animal studies, with a primary impact on the PFC, increasing FE. This effect may be supported by the modulation of BDNF and regulation of the GABAergic system within the PFC [297]. Several studies have shown that microinfusion of BDNF into the PFC induces FE [314], affecting conditioned fear memories [170]. Chronic treatment with lurasidone significantly increases FE and emotional regulation in serotonin transporter KO rats but not in wild-type (WT) control animals [297]. At the circuit level, these pharmacological effects are associated with modulation of amygdala–prefrontal–hippocampal networks and stress-responsive dopaminergic and serotonergic pathways. Additional mechanisms, including regulation of GABAergic tone, may further contribute to the normalization of fear-related processing. From a translational perspective, these findings support the view that antipsychotic drugs do not merely reduce psychotic symptoms via dopaminergic blockade but also exert significant effects on emotional learning and fear regulation. This may help explain their partial efficacy on affective and anxiety-related dimensions of schizophrenia, which are increasingly recognized as core components of functional outcomes.

#### 3.6.2. Clinical Evidence

Clinical evidence supports the translational relevance of preclinical findings on antipsychotic modulation of fear-related processing. A study in antipsychotic-naïve patients, using the Tool for Recognition of Emotions in Neuropsychiatric Disorders (TRENDS), reported that risperidone can improve deficits in fear recognition compared to healthy controls, suggesting partial normalization of emotion processing deficits associated with psychosis [303]. Complementing these findings, a randomized prospective trial comparing risperidone and haloperidol during emotional induction paradigms (such as fear, sadness, anger, joy, and disgust) demonstrated differential effects on emotional regulation. Risperidone was associated with reduced physiological reactivity and reduced subjective experience of negative emotions, while preserving responses to positive emotional stimuli. In contrast, haloperidol showed a less balanced modulation of emotional responses. These results suggest that atypical antipsychotics may exert a broader regulatory influence on affective processing compared to typical agents. Neuroimaging evidence further supports these differences at the circuit level. Patients treated with long-acting risperidone injections showed enhanced amygdala reactivity to fearful faces compared to those receiving conventional antipsychotics, while the latter group exhibited increased ventromedial prefrontal cortex (vmPFC) activation in response to fear expressions. These findings indicate that different antipsychotic classes may differentially modulate the amygdala–prefrontal circuitry underlying fear processing [304]. Overall, clinical evidence converges with preclinical findings in suggesting that atypical antipsychotics exert a more robust modulation of fear-related emotional processing and corticolimbic activity compared to typical agents. In conclusion, clinical findings suggest that atypical antipsychotics exert a more pronounced modulatory effect on emotional processing and fear-related neural responses compared to typical agents, likely through differential regulation of striatal dopamine D2R blockade and serotonergic tone. These effects are reflected in altered amygdala–prefrontal activity and improved emotional regulation, supporting a translational correspondence with preclinical models of fear modulation in schizophrenia. In addition to pharmacological approaches aimed at restoring neurotransmitter balance and normalizing fear-related circuitry, psychological interventions represent an alternative and complementary strategy to address aberrant fear processing in schizophrenia.

### 3.7. Psychotherapy and Aberrant Fear in Schizophrenia Patients

Increasing evidence has shown that different psychotherapeutic approaches, such as cognitive–behavioral therapy (CBT), family therapy, and metacognitive training, can be supportive in addition to antipsychotic treatment to improve symptoms in schizophrenia patients [315,316,317,318,319]. Psychotherapeutic treatments can also improve emotional dysregulation, including fear, and the subsequent worsening of symptomatology [320,321]. Evidence supported the hypothesis that psychotherapies, particularly CBT, can modulate the epigenetic structure in patients with psychiatric disorders. These data suggest a potential role of such treatment methodologies in schizophrenia with altered levels of monoamine oxidase A (MAOA) promoter methylation, particularly at cytosine–guanine (CpG) sites [322,323]. The relationship between emotional dysregulation, such as fear, and some psychiatric disorders is well established [324]. In this context, some neuroimaging studies conducted on patients with bipolar disorder or schizophrenia have demonstrated normalization or enhancement of signals in various brain regions (e.g., the dorsolateral PFC, posterior cingulate cortex, and amygdala) associated with continuous cycles of psychotherapy. Specifically, the results of a facial expression task after psychotherapy showed an increase in the fMRI signal in dorsolateral PFC activation and a decrease in amygdala activation with symptom improvement [324,325]. A clinical study showed that patients with schizophrenia exhibited a reduction in aberrant emotions (such as fear and anger) throughout 4-month combinate psychotherapy, including family group, psychodynamic, supportive, and psychoeducational psychotherapy [326]. A retrospective clinical study conducted on 40 patients diagnosed with schizophrenia treated with antipsychotic drugs reported that cognitive therapy resulted in an improvement in hallucinatory symptomatology, social functioning, quality of life in daily activities, and fear response [327]. An exploratory, insight-oriented psychotherapy has been proposed for patients with schizophrenia [328]. However, this psychotherapy approach could also be efficacious for reversing the aberrant fear of these patients. An innovative psychotherapy approach for patients with schizophrenia is based on virtual reality [329,330]. Specifically, Leff et al. proposed “Avatar Therapy” for treatment-resistant schizophrenia (TRS) patients, involving an immersive virtual reality system in which patients interact with the Avatar, a virtual representation of their main hallucinations controlled and animated by the therapist [331]. In this regard, patients with TRS showed an aberrant fear response during immersive virtual reality with increased interference at the occurrence of primary needs [332]. Problems related to fear and anxiety are among the most prevalent forms of mental illnesses compared to other psychiatric symptoms. Therefore, these findings offered new perspectives for the development of personalized treatments for the management of schizophrenia patients. Psychotherapy could exert its influence on synapses through the modulation of synaptic plasticity and the regulation of neurotransmitter pathways. However, further studies are needed to understand the implications of psychotherapy in neurobiological processes related to aberrant emotional responses to fear. In conclusion, it can be asserted that psychotherapy, when integrated with conventional antipsychotic treatments, holds the potential to yield significant benefits in the management of aberrant fear observed in schizophrenia patients.

Taken together, evidence suggests that psychotherapeutic interventions may ameliorate aberrant fear in schizophrenia by enhancing top-down prefrontal regulation of limbic circuitry, particularly amygdala reactivity, and by promoting adaptive changes in synaptic plasticity and emotional regulation processes. These effects converge on improved salience evaluation and reduce emotional dysregulation across multiple therapeutic modalities.

## 4. Discussion

We have reviewed the putative neurobiological basis of fear processing with the specific aim of tracking, from a translational perspective, the potential role of fear and its modulation in schizophrenia. Although studies comparing aversive conditioning in schizophrenia patients compared to healthy controls have produced mixed results [186,253,254,333,334], several findings point to a subpopulation of schizophrenia patients who exhibit abnormal responses to salient stimuli [55,56,335]. Impaired LI has also been reported in schizophrenia patients [72,336,337]. Notably, LI deficits have also been observed in healthy subjects, as well as in rodents, after the administration of dopamine agonists [338,339], and these deficits are reversed by both first-generation and second-generation antipsychotics [338,339,340]. In this context, functional neuroimaging studies indicate that patients with schizophrenia show altered recognition of fearful faces and display marked limbic hyperactivity even during non-fearful face recognition [341]. A recent meta-analysis further confirmed that individuals with schizophrenia rate pleasant stimuli as less positive and more negative than healthy controls [342].

PET studies indicated altered dopaminergic activity in patients with schizophrenia, including increased tonic dopamine release in the amygdala and ventral striatum [274] and augmented phasic release in response to salient stimuli such as fear [263]. These findings implicate the dopaminergic system in the processing of emotional perception, including fear [4]. Both preclinical and clinical evidence showed that endogenous dopamine release is able to control and facilitate the formation of amygdala-dependent fear memories [13]. Moreover, during FE, activation of nigrostriatal dopamine neurons modified *cFos* expression in the CeA and hippocampal CA1, preventing fear renewal [343]. NMDAR and glutamatergic neurotransmission in the BLA are functionally engaged during FC to regulate neuroplasticity processes [344,345,346,347]. NMDAR are therefore implicated in fear processing, a link that is consistent with the hypothesis of NMDAR hypofunction in schizophrenia pathophysiology [348]. Preclinical studies specifically demonstrated that NMDAR contributes to the acquisition, retrieval, and extinction of fear [33,34], and that blockade of NMDAR subunits (GluN2A and GluN2B) is able to disrupt these learning and conditioning processes [86]. Another consideration is the potential role of CF in synaptic modulation relevant to schizophrenia. For example, exposure to CF has been shown to suppress dopamine release in the *nucleus accumbens* [205], and all brain regions critically involved in fear processing receive massive dopaminergic innervation from the VTA [349,350]. Glutamatergic neurotransmission also modulates fear responses: administration NMDAR antagonists into the lateral and basal amygdala nuclei of the amygdala impairs fear learning [351,352,353]. Both CF paradigms and antipsychotic treatment can alter the expression of postsynaptic density proteins and reorganize dendritic spines [88,89,90,354,355].

Moreover, psychotherapeutic approaches (such as CBT) [315,316] may have, like in severe anxiety disorders [356], a significant impact on fear-induced emotional dysregulation and aberrant salience in schizophrenia patients in the early stages of even the prodromal phase of the disorder [291,292,293,303]. Despite these advances, several critical gaps in knowledge remain and represent key priorities for future research. First, schizophrenia is characterized by substantial clinical and neurobiological heterogeneity, and it remains unclear whether abnormalities in fear conditioning, latent inhibition, and salience processing are present across the entire diagnostic spectrum or are instead confined to specific biologically or clinically defined subgroups. Second, mechanistic studies integrating multimodal approaches are needed to disentangle whether dopaminergic dysregulation, NMDAR hypofunction, and amygdala–striatal circuit dysfunction represent parallel abnormalities or hierarchically organized pathophysiological drivers of fear-processing alterations. Third, the translational validity of preclinical paradigms such as fear conditioning and latent inhibition in capturing complex human phenomena remains only partially established. Finally, longitudinal and interventional studies are urgently needed to determine whether these alterations reflect stable vulnerability traits or state-dependent phenomena modulated by disease progression and pharmacological or psychotherapeutic interventions. In this context, combined pharmacological and psychotherapeutic strategies may offer a particularly promising avenue for modulating fear-related circuitry and improving functional outcomes.

## 5. Conclusions and Future Perspectives

More investigation into the network-level neurobiological mechanisms underlying aberrant fear processing in psychosis may substantially advance the current understanding of schizophrenia pathophysiology, particularly with respect to the persistent alterations contributing to core affective and salience-related symptoms. In this framework, converging evidence on dopaminergic and glutamatergic dysfunction highlights fear processing as a key translational construct linking circuit-level abnormalities to clinical phenomenology. However, current findings also underscore critical and still unresolved issues, including the marked heterogeneity of fear-related alterations across patients, the incomplete delineation of causal interactions between dopamine and glutamate systems within amygdala–striatal circuits, and the limited translational validity of preclinical fear paradigms in capturing complex human symptoms such as aberrant salience and paranoid ideation. Addressing these gaps through multimodal, longitudinal, and computationally informed approaches will be essential to move from descriptive associations toward mechanistic stratification of psychosis. This may ultimately enable the identification of biologically meaningful subtypes and the development of targeted interventions. In this perspective, a more precise understanding of how antipsychotic treatments and psychotherapeutic interventions modulate fear-related circuitry—and how these effects interact with dopamine sensitization processes—may open new avenues for early, mechanism-based treatment strategies in schizophrenia.

## Figures and Tables

**Table 1:** Summary of cued fear conditioning and contextual fear conditioning reported paradigms. ACC, anterior cingulate cortex; FC, fear conditioning; mPFC, medial prefrontal cortex; SCZ, schizophrenia.

FC Paradigm	Conditioned Stimulus	Threat Dimension Modeled	Main Structures Involved	Relevance to SCZ-Related Fear Processing
Cued fear conditioning (cued FC)	A discrete sensory cue, usually a tone or light, is paired with an aversive unconditioned stimulus.	Behavioral response to an immediate and discrete threat.	Mainly relies on the extended amygdala complex, including amygdala-centered circuits involved in fear learning and expression, like sensory thalamic/cortical inputs.	Preclinical models to investigate how discrete neutral stimuli may acquire aberrant salience and abnormal emotional processing in SCZ.
Contextual fear conditioning (context-FC)	The environmental context in which the aversive unconditioned stimulus occurs acts as the conditioned stimulus.	Behavioral response to a contextual or more remote threat.	Mainly involves the hippocampus, ACC, mPFC, and amygdala.	Preclinical models to investigate contextual fear memory, altered contextual appraisal, fear generalization, and emotional dysregulation relevant to SCZ-like phenotypes.

**Table 3:** Neurotransmission and fear-conditioned animal models in schizophrenia. α(7)-nAChRs, alpha(7)-containing nicotinic acetylcholine receptors; ACC, anterior cingulate cortex; AP-5, D,L-2-amino-5-phosphonopentanoic acid; BLA, basolateral amygdala; CaMKII, calcium–calmodulin-dependent protein kinase II; cAMP, cyclic adenosine monophosphate; context-FC, contextual fear conditioning; D1R, dopamine D1 receptor; D4R, dopamine D4 receptor; DA, dopamine; FC, fear conditioned; FE, fear extinction; GABA, gamma-aminobutyric acid; GRF1, growth-regulating factor 1; HRM, heterozygous reeler mice; KO, knockout; MA, methamphetamine; MAPK, MAP kinase; mGluR2, metabotropic glutamate receptor 2; mGluR3, metabotropic glutamate receptor 3; MK-801, 5-methyl-10,11-dihydro-5H-dibenzo[a,d] cyclohepten-5,10-imine maleate; mPFC, medial prefrontal cortex; NAc, nucleus accumbens; NCAM, neural cell adhesion molecule; NL-2 R215H KI, (neuregulin 2 Arg215 → His215 mutation knock-in; NMDA, N-methyl-D-aspartate; PAMs, positive allosteric modulators; PCP, phencyclidine; PFC, prefrontal cortex; PKA, protein kinase A; PP1, protein phosphatase 1; PPI, prepulse inhibition; SCZ, schizophrenia; 5-HT, 5-hydroxytryptamine; WT, wild-type.

Neurotransmission	Study Design/Model	Outcomes	References
Dopamine	In vivo microdialysis preclinical study/latent inhibition rat model (footshock and tone-conditioned stimuli).	Exposure to tone and footshock inhibited DA release in the NAc envelope and resulted in improved freezing.	[205]
	In vivo and ex vivo preclinical study/amphetamine- and PCP-treated rats.	The mGlu2/3 receptor agonist (LY404039) increased DA and 5-HT release/turnover in the PFC and reduced fear-potentiated startle.	[14]
	Preclinical study/rats (footshock and tone-conditioned stimuli); raclopride and saline administration.	D2R by the increase in the signal-to-noise of IL neurons facilitate FE.	[206]
	Preclinical study/rats (olfactory FC and footshock); ketamine–xylazine administration.	The salience of fear memory is regulated by transmission D4R and depends on CaMKII, cAMP/PKA, and PP1 signaling.	[133]
	Preclinical study/C57BL/6 mice.	D2R in the ACC are required for observational fear learning.	[203]
	Preclinical study/rats (conditioned to footshock); MA, MK-801, amfonelic acid; fluoxetine hydrochloride; and nemonapride were administrated.	MA induces behavioral hypersensitivity to stress and fear and D1, 2, 3, 4 antagonists improved this symptomatology.	[158]
Glutamate	In vivo and in vitro preclinical study/ICR (CD1), mGlu2 and mGlu3 KO mice.	Administration of a selective mGlu3 negative allosteric modulator altered FE in mPFC.	[207]
Dopamine/ Glutamate	Preclinical study/rats (foot shock, dark conditioning box, acoustic startle stimuli); AP-5 and saline.	The BLA can regulate latent fear-enhanced startle inhibition and may play a crucial role in SCZ.	[164]
	Preclinical study/rats (conditioned by tone and footshock); saline or (+)-HA-966 were administrated.	Conditioned fear induced elevation in serum corticosterone and freezing behavior in control animals and resulted in an increase in mPFC of DA and 5-HT.	[159]
	Preclinical study/NCAM−/− mice and WT.	NCAM and PSA expression abnormalities cause deficits in hippocampal synaptic plasticity and context-FC by inhibition of the GluN2B-Ras-GRF1-p38 MAPK signaling.	[208]
	Preclinical study/SynGAP mutant mice (noise tone and footshock stimuli and PPI of acoustic startle responses).	SynGAP mutants exhibit reduced freezing in response to a conditioning tone associated with mild foot-shocking.	[165]
	Preclinical study/C57BL/6 mice, PCP-treated.	Mice with selective impairment of presynaptic plasticity showed alteration in the fear memory.	[166]
	Preclinical study/pregnant dams; postnatal rats; CGP 40116 administration.	NMDA receptor antagonists induced a higher level of fear exhibited.	[209]
	Preclinical study/nicotine, MK-801 and APV administration in C57BL/6J mice.	NMDA receptor antagonist determined a deficit in context-FC that was ameliorated by nicotine.	[210]
	Preclinical study/postnatal male rats; LY379268 or saline administration.	mGluR2/3 agonists have been used in SCZ and for improving memory and fear.	[211]
Serotonin GABA	Preclinical study/rats (light/dark schedule and fed ad lib water and chow); scopolamine or Ro 4368554 are used.	5-HT6 antagonists modulated sensory gating and FC in SCZ.	[204]
	Preclinical study/NL-2 R215H KI mice.	Abnormal GABAergic transmission in mPFC alters the fear response and synaptic transmission in SCZ.	[41]
	Preclinical study haploinsufficient HRM.	GABA turnover rate is decreased in cortex, hippocampus, and striatum of HRM that presented an aberrant response to fear, when compared to WT.	[185]
	Preclinical study/ketamine, saline or bicuculline methobromide are administered in rats.	In the SCZ model, the reduction in GABAergic activity in the PFC exacerbated fear learning.	[190]
	Preclinical study/NCAM mice and WT; treated with amphetamine and MK-801.	NCAM increase in contextual and cued FC mice altered synaptic connectivity of GABAergic interneurons relevant to SCZ.	[191]
	Preclinical study/rats treated with saline and ketamine.	Ketamine administration results in alteration in GABAergic transmission and impairs learning in the emotional conditioning task.	[45]
	Preclinical study/cells in rat amygdala slices.	α(7)-nAChRs enhances GABAergic inhibition, and increases spontaneous inhibitory postsynaptic currents in principal BLA neurons.	[195]
	Preclinical study/deficit model of scopolamine-induced FC in rats: RO5126946 (a PAMs of α7nAChR) was administered.	PAMs have a beneficial effect on fear memory.	[200]

**Table 4:** Summary of reported neuroimaging findings relevant to aberrant fear processing in schizophrenia. ACC, anterior cingulate cortex; BLA, basolateral amygdala; BOLD, blood-oxygenation-level-dependent; CeA, central amygdala; FC, fear conditioning; fMRI, functional magnetic resonance imaging; Glx, glutamate + glutamine; mPFC, medial prefrontal cortex; MRS, magnetic resonance spectroscopy; PET, positron emission tomography; PFC, prefrontal cortex; rCBF, regional cerebral blood flow; SCZ, schizophrenia.

Neuroimaging Approach	Main Brain Regions or Networks Involved	Main Findings	Relevance to Aberrant Fear Processing in SCZ
PET/rCBF studies	ACC, *claustrum*–insular–amygdala region, orbitofrontal cortex, amygdala, ventral *striatum*	PET studies showed increased rCBF in the ACC and *claustrum*–insular–amygdala region during induced anxiety, while orbitofrontal activation was associated with anticipatory anxiety. In SCZ, PET studies reported altered amygdala and ventral striatal activities.	Involvement of limbic and mesolimbic circuits in threat appraisal, anticipatory anxiety, salience attribution, and altered emotional reactivity in SCZ.
fMRI/BOLD studies	Association cortices, ACC, anterior insula, amygdala, mPFC, occipitofrontal cortex, fusiform gyrus, superior temporal sulcus	Human FC-fMRI studies showed increased BOLD responses in association cortices and activation of a central autonomic–interoceptive network involving ACC and anterior insula; amygdala, prefrontal and temporal regions were implicated in the processing of salient stimuli.	Fear processing in humans depends on distributed cortical–subcortical networks correlated to aberrant fear in SCZ.
Functional connectivity studies	Salience network, default network, anterior insula, dorsomedial frontal cortex, frontal and parietal cortices	Fear generalization studies showed activation of the salience network and deactivation of the default network in healthy subjects; reduced salience network activation and impaired default network deactivation were reported during fear generalization in SCZ.	Abnormal discrimination between threatening and non-threatening stimuli, fear generalization, and dysregulated salience attribution in SCZ.
MRS studies	Frontal and temporal regions, ACC	Negative emotional response to auditory verbal hallucinations correlated with lower Glx levels in frontal and temporal regions. Reduced Glx in the ACC was proposed to contribute to impaired cognitive control and emotional dysregulation.	Altered glutamatergic tone and impaired top–down control are associated with aberrant fear associated with auditory verbal hallucinations.
Structural MRI studies	Amygdala, *hippocampus*, ACC	Structural MRI evidence showed bilateral amygdala volume reduction in SCZ, while first-episode studies reported hippocampal volume reduction more consistently than amygdala alterations.	Structural corticolimbic abnormalities, altered emotional processing, fear learning, and disease-stage-dependent changes are reported in SCZ.
Amygdala functional and structural findings	Amygdala, BLA, CeA, subgenual ACC, PFC, ventral *striatum*	SCZ studies reported reduced right amygdala activity during emotional processing, left amygdala hyperactivity associated with positive symptoms, altered amygdala–subgenual ACC coupling, and abnormal limbic responses to salient stimuli.	Impaired amygdala–prefrontal connectivity and altered limbic dopaminergic modulation may underlie defective emotion recognition, hyperarousal, aberrant salience, and fear experiences in SCZ.

**Table 5:** Clinical and preclinical evidence on antipsychotic drugs in fear and schizophrenia studies. Abbreviations: 5-HT, 5-hydroxytryptamine; AMI, amisulpride; ARI, aripiprazole; ASE, asenapine; BDNF, brain-derived neurotrophic factor; BOLD, blood-oxygenation-level-dependent; CAR, carbamazepine; context-FC, contextual fear conditioning; CLO, clozapine; CPZ, chlorpromazine; DA, dopamine; FC, fear conditioning; FE, fear extinction; fMRI, functional magnetic resonance; GABA, gamma-aminobutyric acid; HAL, haloperidol; HC, healthy control; LAI, long-acting injection; KO, knockout; LUR, lurasidone; MA, methamphetamine; MK-801, 5-methyl-10,11-dihydro-5H-dibenzo[a,d]cyclohepten-5,10-imine maleate; OLA, olanzapine; PFC, prefrontal cortex; PPI, prepulse inhibition; RIS, risperidone; SCZ, schizophrenia; SERT, serotonin transporter; SHR, spontaneously hypertensive rat; ZIP, ziprasidone.

Antipsychotics	Study Design	Model/Subjects	Outcomes	References
Acute CLO (6 mg/kg)	Preclinical study	C57BL/6J mice, ketamine and medetomidine	Mice with habenula lesions that have a FC showed a decrease in PPI.	[291]
Acute administration of: CLO (20 mg/kg, sc), HAL (0.05 mg/kg, sc), CPZ (10 mg/kg, ip) and OLA (0.5, 1.0, and 2.0 mg/kg, sc)	Preclinical study	Rats	HAL has little effect on conditioned reactive fear, while CLO and OLA have inhibitory effects on fear responses.	[292]
HAL (0.5 mg/kg), AMI (50 mg/kg), ZIP (2 mg/kg), CAR (30 mg/kg), RIS (0.5 mg/kg), CLO (2.5 mg/kg)	Preclinical study	Normotensive and SHR	The context-FC deficit presented by SHRs is reversed by neuroleptic drugs.	[36]
Acute ARI (0.1–10 mg/kg)	Preclinical study	Rats	ARI is effective for the reduction in freezing.	[293]
CLO (5 mg/kg i.p.)	Preclinical study	Neonatal MK-801 rats (pups)	CLO blocked the increase in PPI induced by FC and reduced startle amplitude.	[294]
CLO (1, 3 or 10 mg/kg), HAL (1 mg/kg)	Preclinical microdialysis study	MA-treated rats	CLO is superior to HAL in the regulation of DA release in the amygdala.	[295]
HAL (0.05 mg/kg)	Preclinical study	Rotenone neonatal rats	Rotenone resulted in hyperlocomotion, reduced social interaction, and decreased response to context-FC; this phenotype is reverted by HAL.	[296]
Chronic LUR (10 mg/kg)	Preclinical study	SERT KO mice	LUR may improve emotional increasing FE by BDNF and GABA modulation in the PFC.	[297]
OLA (1–10 mg/kg), CLO (0.3–10 mg/kg), raclopride (3–30 mg/kg), HAL (3 mg/kg), spiperone (0.1–1 mg/kg), nemonapride (1 mg/kg) and CPZ (3–30 mg/kg)	Preclinical study	Rats	CLO decreased conditioned freezing. OLA, HAL, spiperone, nemonapride, and raclopride also inhibited conditional freezing.	[298]
Perospirone (0.3–3 mg/kg p.o.), CLO (1–30 mg/kg, p.o.), RIS (0.03–1 mg/kg, p.o.), ritanserin (0.1–1 mg/kg, p.o.), ketanserin (0.3–1 mg/kg, p.o.), HAL (0.1–3 mg/kg, p.o.), CPZ (3–100 mg/kg, p.o.)	Preclinical study	Rats	Perospirone, CLO, RIS, ritanserin, and ketanserin ameliorated freezing induced by CF.	[298]
Chronic RIS (0.5 mg/kg) and HAL (0.15 mg/kg)	Preclinical study	Rats	RIS and HAL do not improve behavioral alterations caused by kainic acid.	[299]
ARI (10 mg/kg) and HAL (1 mg/kg)	Preclinical study	MA-sensitized rats	ARI reduced both tonic and phasic DA levels in the amygdala of FC rats. HAL only decreased the phasic release, and increased the extracellular DA in the amygdala	[300]
Acute ASE (0.3 mg/kg), CLO (1–10 mg/kg), and OLA (0.3–3 mg/kg)	Preclinical study	Rats	The alteration on behavior of FC rats (using footshock) is reversed by antipsychotics drugs.	[301]
RIS, HAL	Randomized and prospective clinical study	30 patients with SCZ (following a wash-out period of at least 1 week for prior oral antipsychotic treatment)	Atypical antipsychotics were more effective and efficacious than typical in emotional regulation with increased 5-HT and DA in the PFC.	[302]
RIS	Longitudinal clinical study	30 HC and 25 antipsychotic-naïve SCZ patients	RIS improved fear recognition deficits in SCZ patients.	[303]
RIS LAI, HAL decanoate, pipotiazine palmitate, flupentixol and fluphenazine decanoate	Controlled clinical trial	16 SCZ patients treated with RIS LAI, 16 SCZ patients treated with conventional LAI and 16 HC.	In patients treated with RIS LAI, the BOLD fMRI signal in the left amygdala in response to fearful faces is greater than patients treated with conventional antipsychotics.	[304]

## Data Availability

No new data were created or analyzed in this study.

## Abbreviations

The following abbreviations are used in this manuscript:

fMRI	Functional magnetic resonance imaging
PET	Positron emission tomography
mPFC	Medial prefrontal cortex
FC	Fear conditioning
CS	Conditional stimulus
CR	Conditional response
ACC	Anterior cingulate cortex
tFC	Trace fear conditioning
D1R	D1 dopamine receptor
D2R	D2 dopamine receptor
NMDAR	N-Methyl-D-aspartic acid receptors
AMPAR	α-amino-3-hydroxy-5-methyl-4-isoxazplepropionic acid receptor
SHR	Spontaneous hypertensive rats
SGA	Second-generation antipsychotics
GIT1	GPCR-kinase interacting protein 1
KO	Knockout
NRXN-β	β-neurexin
SH3	SRC homology 3 domain
SHANK	Multiple ankyrin repeat domains 3
GABA	γ-aminobutyric acid
TCF4	Transcription factor 4
PPI	Prepulse inhibition
BLA	Basal lateral amygdala
NRG1	Neuregulin 1
PFC	Prefrontal cortex
FE	Fear extinction
PTSD	Post-traumatic stress disorder
vmPFC	Ventromedial prefrontal cortex
DCS	D-Cycloserine
MK-801	5-methyl-10,11-dihydro-5H-dibenzo[a,d]cyclohepten-5,10-imine maleate
mTORC	Mammalian Target of Rapamycin complex 1
LI	Latent inhibition
Nlgn 2	Neuroligin-2
KI	Knock-in
LTP	Long-term potentiation
LTD	Long-term depression
PSD	Postsynaptic density
PKB	Protein kinase B
TrkB	Tropomyosin receptor kinase
Shc	Mitochondrial adaptor p66
MAPK	Mitogen-activated protein kinase
HAB	High anxiety-related behavior
NAB	Normal anxiety-related behavior
pCaMKII	Phosphorylated calcium–calmodulin-dependent protein kinase II
mTOR	Mammalian target of rapamycin
GluR1	Glutamate receptor 1
CACNA1C	Voltage-dependent L-type calcium channel subunit alpha-1C
VTA	Ventral tegmental area
FPS	Fear-potentiated startle
CeA	Central amygdala
IPC	Intercalated paracapsular islands
MSN	Medium-sized spiny neuron
mGluR	Metabotropic glutamate receptor
ROIs	Regions of interest
rCBF	Regional cerebral blood flow
BOLD	Blood-oxygenation-level-dependent
SN	Salience network
DN	Default network
AVHs	Auditory verbal hallucinations
Glx	Glutamate + glutamine
IAPS	International Affective Picture System
5-HT2R	5-hydroxytryptamine 2 receptor
SDA	Serotonergic and dopaminergic antagonist
DRN	Dorsal raphe nucleus
BDNF	Brain-derived neurotrophic factor
WT	Wild-type
TRENDS	Recognition of Emotions in Neuropsychiatric Disorders
CBT	Cognitive–behavioral therapy
MAOA	Monoamine oxidases A
TRS	Treatment-resistant schizophrenia
FGA	First-generation antipsychotic
